# Identification and Functional Assignment of Genes Implicated in Sperm Maturation of Tibetan Sheep

**DOI:** 10.3390/ani13091553

**Published:** 2023-05-06

**Authors:** Taotao Li, Huihui Wang, Ruirui Luo, Huibin Shi, Manchun Su, Yi Wu, Qiao Li, Keyan Ma, Yong Zhang, Youji Ma

**Affiliations:** 1 College of Animal Science and Technology, Gansu Agricultural University, Lanzhou 730070, China; litt@gsau.edu.cn (T.L.);; 2 Gansu Key Laboratory of Animal Generational Physiology and Reproductive Regulation, Lanzhou 730070, China; 3 Animal Husbandry, Pasture and Green Agriculture Institute, Gansu Academy of Agricultural Sciences, Lanzhou 730070, China

**Keywords:** Tibetan sheep (*Ovis aries*), epididymis, sperm maturation, RNA sequencing, differential expression

## Abstract

**Simple Summary:**

Tibetan sheep are the characteristic sheep breed on the Qinghai-Tibet Plateau, characterized by good adaptability to the hypoxic conditions, delayed maturation, and low fecundity. The epididymis is a male reproductive organ well known to be responsible for sperm transport, storage, and maturation, which is crucial for male fertility. To clarify the dynamic gene expression patterns and their potential contribution during sperm maturation of Tibetan sheep, in this study we characterized the comprehensive transcriptional profiles in the three epididymal areas (caput, corpus, and cauda) of Tibetan sheep using RNA sequencing. The results revealed that numerous genes are present in Tibetan sheep epididymis in a stage-region-dependent manner, showing more dramatic changes in gene expression in various epididymal areas of post-pubertal Tibetan sheep. These genes perform some sort of function in reproduction, development and morphogenesis, and immune privilege to facilitate the maturation of spermatozoa and to provide the microenvironment required for sperm development and maturation. This study provides new insights into the molecular mechanisms by which genes are regulated during post-testicular sperm development.

**Abstract:**

While traveling through the epididymis, immature sheep spermatozoa undergo a sequence of processes that ultimately give them the capacity to swim and fertilize an egg. Different gene expression patterns may be found in the epididymal caput, corpus, and cauda, conferring variant or unique biological roles during epididymis development and sperm maturation. To search for candidate genes associated with ovine sperm maturation and assess their possible modulating mechanisms, we characterized gene expression in each epididymal segment derived from pre- and post-pubertal Tibetan sheep by RNA sequencing. Compared with pre-puberty, 7730 (3724 upregulated and 4006 downregulated), 7516 (3909 upregulated and 3607 downregulated), and 7586 (4115 elevated and 3471 downregulated) genes were found to be differentially expressed in the post-pubertal caput, corpus, and cauda epididymis, respectively, and real-time quantitative PCR verified the validity of the gathered expression patterns. Based on their functional annotations, most differential genes were assigned to the biological processes and pathways associated with cellular proliferation, differentiation, immune response, or metabolic activities. As for the post-pubertal epididymis, 2801, 197, and 186 genes were specifically expressed in the caput, corpus, and cauda, respectively. Functional annotation revealed that they were mainly enriched to various distinct biological processes associated with reproduction (including the caput binding of sperm to the zona pellucida; fertilization in the caput and corpus; and meiosis in the caput and cauda) and development (such as cell differentiation and developmental maturation in the caput; cell proliferation and metabolism in the corpus; and regulation of tube size and cell division/cell cycle in the cauda). Additionally, we focused on the identification of genes implicated in immunity and sperm maturation, and subsequent functional enrichment analysis revealed that immune-related genes mainly participated in the biological processes or pathways associated with the immune barrier (such as JAM3 and ITGA4/6/9) and immunosuppression (such as TGFB2, TGFBR1, TGFBR2, and SMAD3), thus protecting auto-immunogenic spermatozoa. Additionally, sperm maturation was mostly controlled by genes linked with cellular processes, including cell growth, proliferation, division, migration, morphogenesis, and junction. Altogether, these results suggest that most genes were differentially expressed in developmental epididymal regions to contribute to microenvironment development and sperm maturation. These findings help us better understand the epididymal biology, including sperm maturation pathways and functional differences between the epididymal regions in Tibetan sheep and other sheep breeds.

## 1. Introduction

Spermatozoa in mammals are unable to fertilize an egg once they leave the testis because they are immobile and lack the ability to undergo capacitation. Spermatozoa only fully develop when traveling through the epididymis, including their acquisition of motility and fertilizing capacity [1]. The epididymis joins the testis and vas deferens by a single, highly coiled, convoluted tubule. Principal, basal, narrow, transparent, and halo cells, among others, make up the epididymal epithelial lining, which regulates sperm maturation prior to fertilization, exhibiting a variety of biological roles such as promoting structural development, sensing and responding to changes in their surrounding microenvironment, and communicating with spermatozoa [2]. Before being released during ejaculation, spermatozoa are transported sequentially through the caput, the corpus, and finally the cauda [3,4]. Since spermatozoa tend to be synthetically inactive, maturation relies on the spermatozoa interacting with proteins made and secreted by particular parts of the epididymal epithelium [2]. Exposure to the luminal environment of the epididymis, rather than events inherent to germ cells, is responsible for spermatozoa maturation [5,6]. Although the epididymis seems to be a simple tubule, each segment has its own unique gene expression profile and physical characteristics, which establish and maintain the constantly changing luminal environment required for sperm maturation [7]. Segment-specific secretion of proteins into the luminal fluid of the epididymal epithelium determines gene expression patterns that directly or indirectly impact sperm maturation, transport, and storage [8,9]. The distinct patterns of gene expression in each of these areas have been linked to physiological processes that play a role at various stages of sperm development [10]. Therefore, it is crucial to comprehend epididymal sperm maturation by finding and understanding the role of segment-specific genes. Many genes have been reported to participate in the development and function of mammalian epididymis, such as aquaporins, beta defensins, claudins, cadherins, lipocalins, and the glutathione peroxidase family [11,12]. For instance, GPX5, an important member of the glutathione peroxidase family, is specifically expressed in the mammalian epididymis and functions to protect sperm from reactive oxygen species and lipid peroxidation damage during maturation [13]. Peroxiredoxins (PRDXs) are involved in the protection of sperm function and DNA integrity during epididymal maturation to ensure male fertility [14].

Overall, understanding the maturation of sperm requires a full understanding of the expression of genes and the biological activities they serve in the epididymal segment. Both extrinsic (such as nutritional levels) and intrinsic (such as androgens) factors can affect the production, development, maturation, and quality of sperm through controlling the expression of genes [15,16,17,18]. In order to produce the appropriate luminal environment for the functional development and protection of spermatozoa, greater emphasis must be placed on the regional expression of epididymal genes. Unfortunately, very few reports in this context are available on sheep. Recognizing the regulatory and functional differences in genes between the various parts of the sheep epididymis requires a systematic approach to understanding their transcriptional patterns. Throughout the Qinghai–Tibet Plateau, the remarkable domestic sheep breed known as Tibetan sheep (*Ovis aries*) is found mostly at elevations of 3000 m above sea level [19]. Due to their long-term, excessive dependency on grazing without supplemental food, Tibetan sheep have late sexual maturation (around 1 year old) and a low reproduction rate (including seasonal estrus, initial mating aged 2.5 years old, lambing once per year, and having only 1 lamb at a time,). It is crucial to the study of sheep reproduction to get a deeper understanding of the processes governing epididymal development in male Tibetan sheep. However, the gene profiles in developmental Tibetan sheep epididymis and their function in each epididymal region are unknown. Therefore, we investigated the dynamic gene expression profiles in developmental Tibetan sheep epididymis (caput, corpus, and cauda) using high-throughput RNA sequencing (RNA-seq) technologies and then assessed their potential functions during sperm maturation. The findings of this study have significant implications for understanding the mechanisms regulating ram sperm maturation and, ultimately, for enhancing the quality of semen and, by extension, male reproductive success.

## 2. Materials and Methods

### 2.1. Ethical Statement

All experiments were conducted in accordance with the National Laboratory Animal Welfare instructions (2006-398) and were authorized by the Ethics Committee of the Laboratory Animal Center at Gansu Agricultural University (GSAU-Eth-ASF2022-008).

### 2.2. Sample Collection and Processing

The Xike Tibetan Sheep Breeding Base (Xiahe, China) offered 8 male Tibetan sheep descended from the same father at 2 reproductive stages: pre-puberty (3 months old, 3M; n = 4; weighed 9.23–10.18 kg; scrotal circumference: 8.4–8.9 cm) and post-puberty (1 year old, 1Y; n = 4; weighed 34.25–36.73 kg; scrotal circumference: 17.6–18.3 cm). All Tibetan sheep were from the same farm under traditional grazing management which meant that the sheep grazed in a fenced pasture all year round with free access to food and water without feed supplements. All animals were sacrificed with an overdose of sodium pentobarbitone (intravenous). Following incision of the scrotum, the testis and epididymis were exposed, and the middle segment from each right epididymal region (caput, corpus, and cauda) was harvested immediately, rinsed in PBS to remove excess blood, quickly placed into a centrifuge tube containing RNA protective agent, immediately put into a liquid nitrogen tank, transported back to the laboratory within 1 h, and stored in a −80 °C refrigerator for RNA extraction.

### 2.3. RNA Extraction, Library Preparation, and Sequencing

Total RNA was isolated from frozen epididymal tissue following the manufacturer’s instructions using a Trizol kit (Invitrogen, Carlsbad, CA, USA). RNase-free agarose gel electrophoresis, the Agilent 2100 Bioanalyzer (Agilent Technologies, Palo Alto, CA, USA), and a NanoDrop Spectrophotometer (NanoDrop Technologies, Wilmington, DE, USA) were used to evaluate the RNA quality. All RNA samples had good integrity (RNA integrity number (RIN) > 7.5). The mRNA was purified and fragmented to approximately 200 bp from the isolated total RNA after removal of rRNA, followed by being subjected to first- and second-strand cDNA synthesis. Final cDNA libraries were made by purifying and enriching cDNA fragments that had previously undergone end repair and adaptor ligation before being sequenced using an Illumina HiSeq 2500 platform (Gene Denovo Biotechnology, Guangzhou, China). Finally, we constructed 24 cDNA libraries (4 replicates per group), including pre-pubertal caput (i.e., Cp_3M-1, Cp_3M-2, Cp_3M-3, Cp_3M-4), corpus (i.e., Cr_3M-1, Cr_3M-2, Cr_3M-3, Cr_3M-4), and cauda (Cu_3M-1, Cu_3M-2, Cu_3M-3, Cu_3M-4), as well as post-pubertal caput (i.e., Cp_1Y-1, Cp_1Y-2, Cp_1Y-3, Cp_1Y-4), corpus (i.e., Cr_1Y-1, Cr_1Y-2, Cr_1Y-3, Cr_1Y-4), and cauda (i.e., Cu_1Y-1, Cu_1Y-2, Cu_1Y-3, Cu_1Y-4).

### 2.4. Differential Expression Analysis and Data Processing

High-quality clean data were obtained by filtering low-quality reads and adaptors of all the samples for subsequent analyses. Reference genome assembly Oar v4.0 for sheep was used to align the clean reads utilizing HISAT default parameters. The expression abundances of genes were measured in FPKM using StringTie [20]. DESeq2 was used for group-specific differential expression analysis. Parameters of absolute fold change (FC) ≥ 2 and false discovery rate (FDR) below 0.05 were used to identify differentially expressed genes (DEGs).

### 2.5. Evaluation of Functional Enrichment

In order to better understand the biological functions of DEGs, we conducted a functional enrichment analysis utilizing the GO (http://geneontology.org/ (accessed on 17 September 2022)) and KEGG databases (http://www.genome.jp/kegg/ (accessed on 17 September 2022)).

### 2.6. Quantitative Real-Time PCR as an RNA-Seq Validation Tool

To evaluate the repeatability and reproducibility of RNA sequencing data, a quantitative real-time PCR (qPCR) study was carried out on DEGs. Specifically, cDNA was reverse-transcribed from isolated total RNA using the EvoM-MLV RT Kit (Accurate Biotechnology, Hunan, China), and qPCR assay was carried out using the SYBR Green qPCR Kit (Accurate Biotechnology, Hunan, China). At least three independent repetitions of each experiment were performed. Table S1 contains information on the primers used. Using a 2^−ΔΔCt^ method, the expression levels relative to the internal reference gene (β-actin) were calculated.

## 3. Results

### 3.1. Overview of Transcriptome Data

In all, 187.57 Gb of clean data were generated by the sequencing. The total size of the clean data for each sample was 6.39 Gb, and the Q30 base percentage was 90.13% or higher. Each sample’s clean reads were sequence-aligned to a predefined reference genome, and the alignment accuracy ranged from 87.11% to 89.5% (Table S2).

### 3.2. Differentially Expressed Gene Analysis and Functional Analysis

#### 3.2.1. Identification and Functional Analysis of Differential Expression Genes in Caput Epididymis before and after Puberty

**Figure 1:**
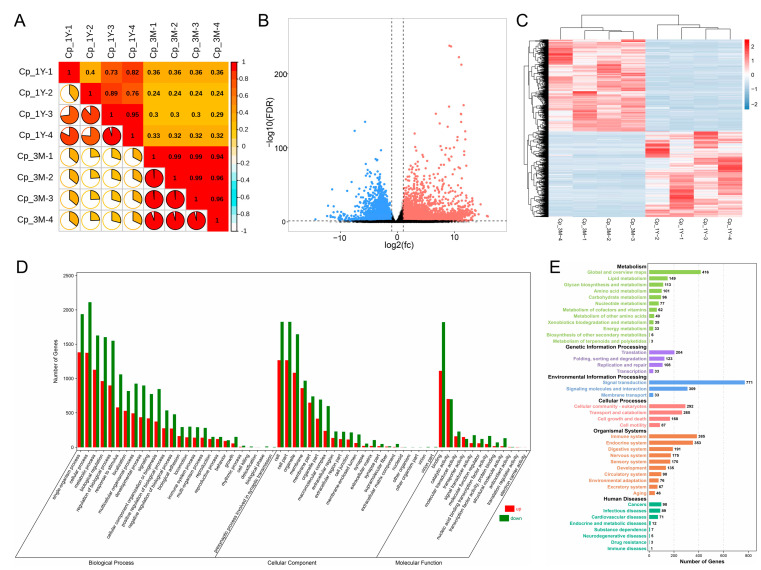
Differential expression and functional analysis of genes in the caput epididymis during the pre- and post-pubertal periods. (**A**) Sample correlation heatmap. (**B**) Volcano plot and (**C**) clustering heatmap for differentially expressed transcripts. (**D**) GO annotation and (**E**) KEGG pathway enrichment analysis for differential genes. Cp_3M and Cp_1Y correspond to caput epididymis from three-month-old and one-year-old group, respectively.

It was found that there was high repeatability across four independent samples from each group, as seen by the sample correlation heatmap derived from the mRNA expression profiles (Figure 1A). A total of 7730 transcripts were expressed differentially with 3724 upregulated and 4006 downregulated in the 1-year-old group (Figure 1B,C). The predominant enriched GO terms of these genes, in the biological process, were associated with metabolism, growth and development, reproduction, biological adhesion, and immune function; in the cellular component, were associated with extracellular region, membrane part, and cell junction; in the molecular function, they were associated with binding, catalytic activity, and transporter activity (Figure 1D). The KEGG analysis revealed that the majority of these differential genes were enriched in metabolic pathways (such as lipid, amino acid, and energy metabolism), cellular processes (such as cellular growth, death, and motility), and organismal systems (such as development, immune, and endocrine systems) (Figure 1E).

#### 3.2.2. Identification and Functional Analysis of Differential Expression Genes in Corpus Epididymis before and after Puberty

**Figure 2:**
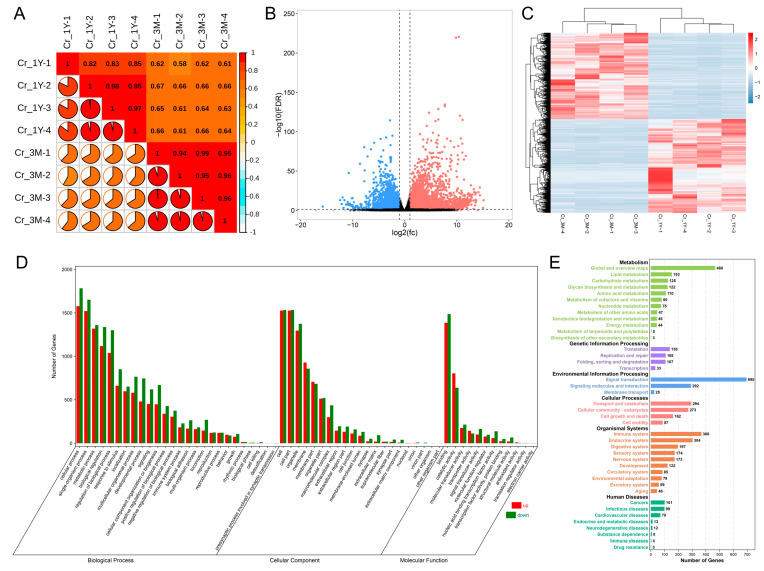
Differential expression and functional analysis of genes in the corpus epididymis during the pre- and post-pubertal periods. (**A**) Sample correlation heatmap. (**B**) Volcano plot and (**C**) clustering heatmap for differentially expressed genes. Differential up- and downregulation are highlighted in red and blue, respectively. (**D**) GO annotation and (**E**) KEGG pathway analysis for differentially expressed genes. Cr_3M and Cr_1Y denote corpus epididymis from three-month-old and one-year-old group, respectively.

The sample correlation heatmap indicated high repeatability of the data from each group (Figure 2A). Compared with the 3-month-old group, the 1-year-old group had 7516 DEGs (3909 upregulated and 3607 downregulated) (Figure 2B,C). According to the functional analysis of GO annotation, these genes were shown to be involved in a wide variety of biological activities, including cellular function, metabolism, growth and development, reproduction, and immunity; in terms of the cellular component, GO terms associated with membrane, organelle, complex macromolecules, outside the cell, and cell junction were the most represented; in terms of molecular function, among the most enriched GO terms were “binding,” “catalytic,” “transporter,” and “molecular function regulator” (Figure 2D). Analysis of these DEGs revealed that they were abundant in metabolic pathways such as lipid metabolism, amino acid metabolism, and energy metabolism, with biological processes such as cell proliferation, death, and motility, as well as pathways associated with development and immune signaling (Figure 2E).

#### 3.2.3. Analysis of Pre- and Post-Pubertal Cauda Epididymal Gene Expression Differences and Their Functional Significance

**Figure 3:**
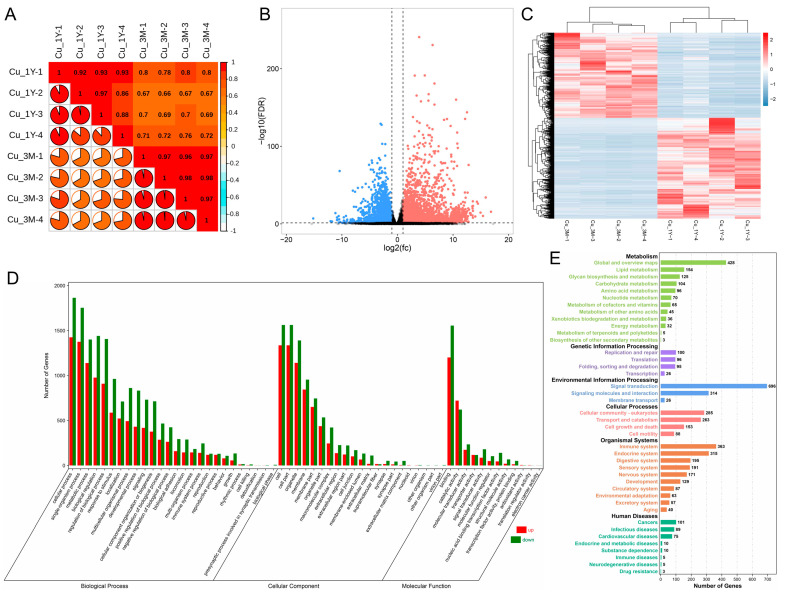
Differential expression and functional analysis of genes in the cauda epididymis during the pre- and post-pubertal periods. (**A**) Sample correlation heatmap. (**B**) Volcano plot and (**C**) clustering heatmap showing differential gene expression between Cu_3M and Cu_1Y groups. (**D**) GO terms and (**E**) KEGG pathways in differentially expressed gene set enrichment analysis. Cu_3M and Cu_1Y represent cauda epididymis from three-month-old and one-year-old group, respectively.

Correlation heatmaps based on gene expression patterns demonstrated high repeatability across four samples from each group (Figure 3A). Differential expression analysis identified a total of 7586 DEGs (4115 elevated and 3471 downregulated) (Figure 3B,C). GO analysis indicted that metabolism, biological adhesion, growth and development, reproduction, and immune function were the main biological processes enriched by genes; with respect to cellular component, almost all gene expression was found within the organelle, outside the cell, membrane part, and cell junction; with respect to molecular function, most genes served some sort of binding, catalytic, or transporter function (Figure 3D). The majority of genes were enriched for their participation in cell proliferation, cell motility, development, immunity, and metabolism (such as lipid, energy, amino acid, and carbohydrate metabolism) (Figure 3E), as determined by the KEGG database.

#### 3.2.4. Region-Specific Gene Expression and Functional Analysis

**Figure 4:**
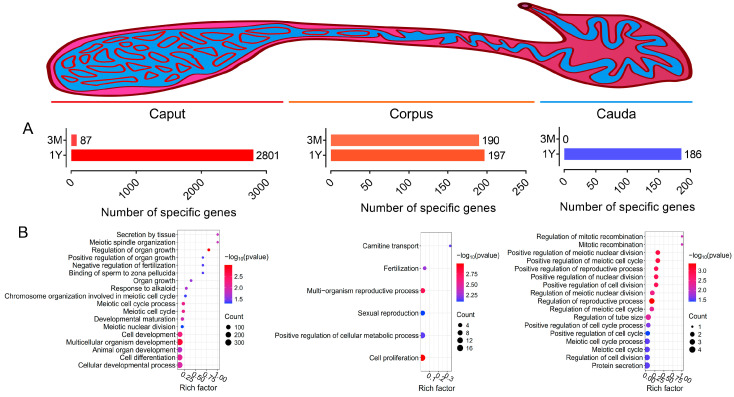
Identification of region-specific genes and their functional analysis. (**A**) Bar graph showing the number of specifically expressed genes in each epididymal region. (**B**) GO annotation—biological processes enriched for the specific genes in post-pubertal caput, corpus, or cauda.

To compare the differences in gene expression and their biological functions across epididymal regions, we searched for genes that were specifically expressed in a single region. Only transcripts with an average FPKM value greater than 1 were considered expressed. For the pre-pubertal epididymis, we found that 87 and 190 genes were specifically expressed in the caput and corpus, respectively, but showed very low expression for the vast majority of genes (FPKM < 2); for the post-pubertal epididymis, 2801, 197, and 186 genes were specifically found in the caput, corpus, and cauda, respectively (Figure 4A). The subsequent GO annotation based on biological processes showed that the genes in the post-pubertal caput were mainly involved in processes associated with reproduction (such as meiosis-associated processes, sperm attachment to the zona pellucida, and the negative control of fertilization) and growth and development (such as multicellular organism development, cell development, cell differentiation, developmental maturation, and organ growth/development); the genes in the post-pubertal corpus were significantly enriched in biological processes including fertilization, sexual reproduction, the reproductive process, cell proliferation, the cellular metabolic process, and carnitine (a nutritional aid for improving sperm motility) transport; the genes in the post-pubertal cauda were significantly enriched in processes associated with reproduction (such as the reproductive process and meiosis), development (regulation of tube size, cell division, mitotic recombination, and cell cycle), and protein secretion (Figure 4B).

#### 3.2.5. Screening and Functional Analysis of Epididymal Immunoprotection-Related Genes

**Figure 5:**
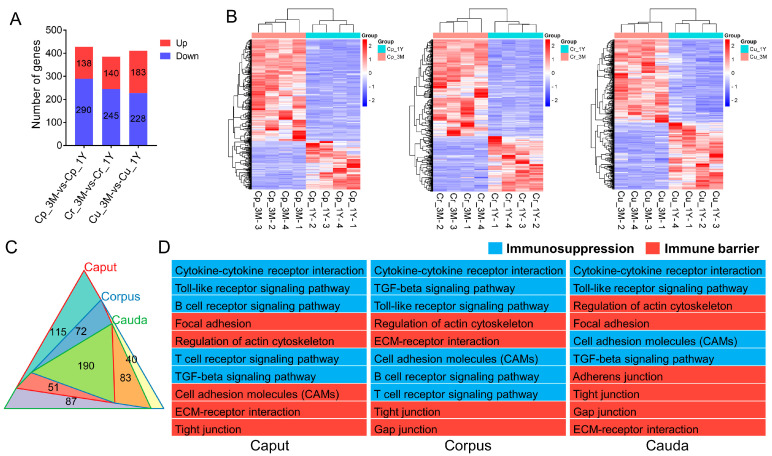
Expression analysis and functional annotation of differential genes related to immunity. (**A**) The histogram and (**B**) clustering heatmap showing the numbers and expression profiles of immune-related genes. (**C**) Venn diagram showing the shared and unique genes in different regions of the epididymis. (**D**) Top 20 significantly enriched KEGG pathways closely related with immunity.

The intraluminal compartment of the epididymis is immunoprotected, similar to the testis. To investigate the potential activities of genes involved in sustaining immune function during epididymal development, we examined the immune-related DEGs in further detail based on the results from the GO and KEGG functional analysis. We discovered 428 (138 elevated; 290 downregulated), 385 (140 upregulated; 245 downregulated), and 411 (183 elevated; 228 downregulated) immune-related differential genes in the epididymis caput, corpus, and cauda, respectively (Figure 5A). Heatmap analysis of these genes revealed remarkable repeatability and gene expression levels across age groups (Figure 5B). See Table S3 for a complete list of these genes and their expression levels. Among these, 190 DEGs were co-expressed in all epididymal regions detected (Figure 5C). In order to better understand and interpret the possible biological roles of the genes, the KEGG database was used to determine the gene–pathway associations for all the immune-related genes across the various comparison groups. These genes were found to be significantly enriched in pathways related to the immune barrier (such as the tight junction, gap junction, adherens junction, ECM–receptor interaction, and modulation of actin cytoskeleton) and immunosuppression (such as cytokine–cytokine receptor interaction, B/T cell receptor signaling pathway, and Toll-like receptor signaling pathway) (Figure 5D).

#### 3.2.6. Analysis of DEGs Associated with Sperm Maturation

**Figure 6:**
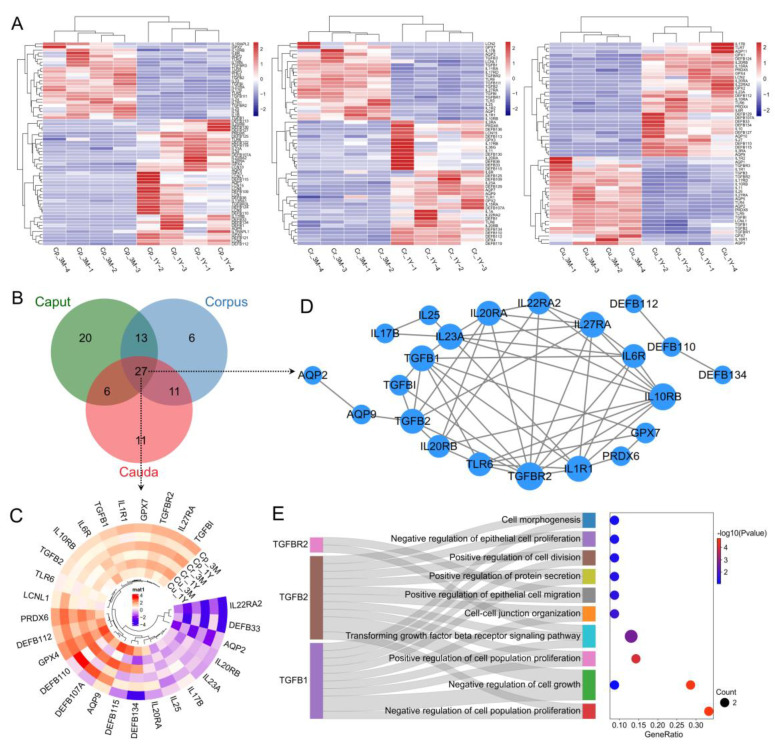
Expression and functional analysis of sperm-maturation-related genes. (**A**) Gene expression heatmap representation of different epididymal segments. (**B**) Venn analysis showing the number of unique and shared genes. (**C**) Tissue expression profiles and (**D**) interaction network of the shared genes. (**E**) The gene–biological process network based on GO annotation of crucial genes in the interaction network.

To probe the potential functions of the genes implicated in sperm maturation, we conducted further analysis of the genes encoding eight kinds of sperm-maturity-related protein families: glutathione peroxidases (GPXs), β-defensins (DEFBs), lipocalins (LCNs), interleukins (ILs), Toll-like receptors (TLRs), transforming growth factors (TGFs), peroxiredoxins (PRDXs), and aquaporins (AQPs). In total, we obtained 66 (41 upregulated and 25 downregulated), 57 (35 upregulated and 22 downregulated), and 55 (31 upregulated and 24 downregulated) differential genes in the caput, corpus, and cauda (Figure 6A), respectively. Of these, 27 genes were co-expressed in all epididymal segments (Figure 6B), with upregulated expression in all post-pubertal segments for most genes (Figure 6C). Interaction network analysis for co-expressed protein-coding gene sets showed that TGFs (TGFB1/2) and their receptor (TGFBR2) were in the more central position of the network (Figure 6D). Further functional annotation revealed the participation of these genes in various cell events, including cell growth, proliferation, division, migration, morphogenesis, and intercellular junction (Figure 6E).

### 3.3. qPCR Validation

**Figure 7:**
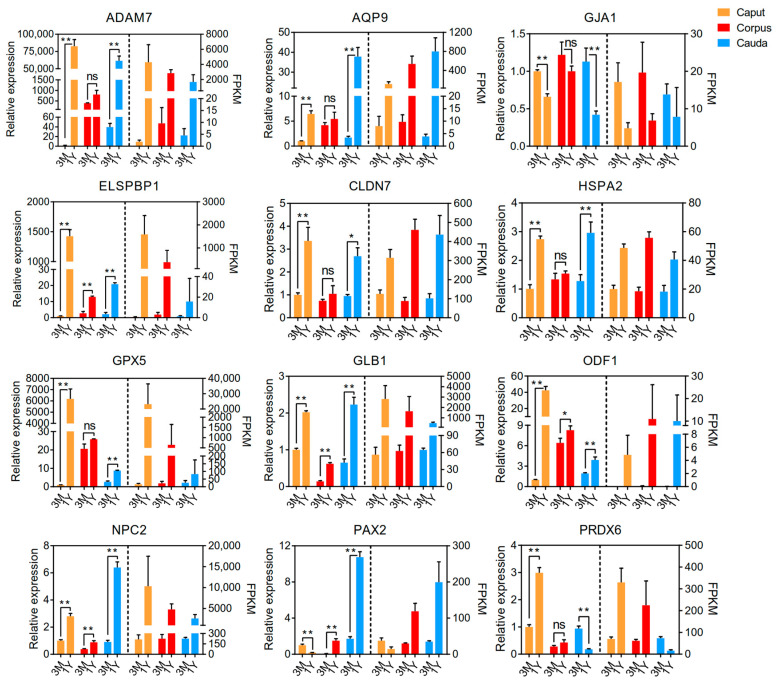
Verification of RNA-Seq results by qPCR. Data represent the mean ± SD. Significance levels: two asterisks, *p* < 0.01; one asterisk, *p* < 0.05; ns, not significant.

In order to ensure the validity of the RNA-seq data, qPCR analysis was performed on 12 randomly chosen DEGs. In general, the qPCR data and RNA sequencing data showed similar expression patterns of elevation or reduction in mRNA between age groups (Figure 7), suggesting the reliability of the transcriptome data.

## 4. Discussion

The characterization of epididymal transcriptome is an attractive topic because the epididymis is a highly regional organ whose core function is to perform all the biochemical changes responsible for post-testicular sperm maturation and storage. To understand the establishment of epididymis development and sperm maturation, a complete and broad-scale transcriptional perspective of the epididymis and its developmental process is required. Here, we obtained 187.57 Gb of clean sequencing data during postnatal development of Tibetan sheep epididymis, allowing a more comprehensive study of ovine epididymal transcriptome. These findings provide the groundwork for comprehending the environment of sperm maturation by shedding light on the landscape of the epididymal transcriptome and a detailed investigation of transcriptomic alterations during epididymal development.

RNA-seq was performed on pre-pubertal and post-pubertal epididymis tissues (caput, corpus, and cauda) to discover the possible genes involved in epididymal development and sperm maturation. We discovered a total of 23,463 transcripts in developmental epididymis tissues. Of these, 7730 (3724 upregulated; 4006 downregulated), 7516 (3909 upregulated; 3607 downregulated), and 7586 (4115 upregulated; 3471 downregulated) transcripts were variably expressed in the caput, corpus, and cauda of the developing epididymis, respectively. Each segment of the epididymis exhibits different gene expression patterns as well as diverse morphological characteristics, which lend this obviously simple tubule a higher level of functional complexity than could be anticipated [21]. Thus, we also analyzed the regional distribution of the transcripts in the caput vs. corpus, caput vs. cauda, and corpus vs. caput. We found that DE mRNAs were more abundant in the cauda epididymis at pre-puberty and more abundant in the caput epididymis at post-puberty. Interestingly, the differential genes in the corpus and cauda epididymis were occupied by the caput epididymis. The gene expression profiles in the corpus and cauda at post-puberty were not similar at all, but both differed from the caput to a similar degree. This is in contrast to previous studies in the human epididymis. James et al. discovered that the gene expression profiles in the corpus and cauda were remarkably similar [22]. This difference might be due to the fact that each species seems to have developed its own sperm maturation strategy. Studies in rodents confirm that the proximal epididymis (initial segment and caput) is the segment most responsive to intraluminal factors proposed to control downstream gene expression [23]. Our results likewise confirm that the corpus epididymis is the most active region for gene expression.

According to a GO and KEGG database-based functional bioinformatics study, these divergent genes mostly function in processes and pathways associated with cell growth, reproduction, immunity, and metabolism. We found that the most enriched term for differential genes in the developmental caput, corpus, and cauda was cellular process, which falls under the biological process domain. We further discovered in the caput that upregulated (higher expression in the 1Y group) genes such as GPX3 and IL13RA1 were engaged in the cellular process term. GPX3 is involved in the protection of mature sperm from reactive oxygen species damage and catalyzes the degradation of peroxides to which sperm are exposed during the maturation process [24]; multiple processes rely on IL, including the development, activation, proliferation, and control of immune cells [25]. These activities in the caput epididymis work together to ensure proper sperm development at an early stage. The upregulated (relatively higher expression in the post-pubertal cauda) genes that were enriched in the cellular process GO term, such as GPR50, CST3, and LCN2, were reported to be correlated with the following cell components: GPR50 was associated with plasma membrane [26]; CST3 is a member of the cysteine protease inhibitor family [27]; proteases and protease inhibitors serve a functional role in protein processing at sperm plasma membrane levels during maturation [28]; and the LCN family is involved in lipid transport, which has implications for a wide range of biological processes such as the immune response, sperm maturation, and storage [29]. The term “cellular process” is used to describe any activity that occurs at the cellular level [30], which may or may not involve a single cell. For example, cellular communication, which involves several cells yet occurs at the cellular level. The upregulated genes in the corpus such as PRDX6 were identified as playing a role in epididymal cellular processes. The antioxidant effect of PRDX6 is important for the protection of sperm during epididymal maturation [31]. These roles of the upregulated genes enriched in the cellular process term, as indicated above, may be relevant for the proposed function of the epididymis such as the establishment of a luminal environment to promote sperm motility and ovum recognition.

Pseudo-stratified epithelia line the epididymal duct and perform several crucial functions, including forming tight junctions and enforcing a physical barrier. Sperm, critically, have immunogenic properties [32]. The testicular barrier and immunosuppressive factors [33,34] have been shown to sustain a non-inflammatory local steady state, which is necessary for reproductive function. Nevertheless, the epididymis has a less stringent blood–tissue barrier than the testis, and while a variety of immunosuppressive factors have been found in the testis, such mechanisms for epididymal immune privilege have not yet been described [35,36]. The blood–epididymis barrier (BEB) is a membrane structure between the immune system and sperm that blocks interactions between sperm autoantigens and the immune system. It also functions to establish and develop the epididymal luminal environment, which shields developing spermatozoa from the immune system and is beneficial for normal sperm maturation [37]. The fully functional BEB is composed of anatomical, physiological, and immunological barriers [35]. The anatomical barrier is made up of tight junctions to restrict molecules and cells from entering and exiting the lumen, while the immunological barrier consists of different immune components inside and outside the tubule/duct [35]. Antigenic spermatozoa are shielded from an immunological response during sperm maturation, a process that is known to rely heavily on immune processes [38]. Hence, we analyzed immune-related differential genes in the pre- and post-pubertal caput, corpus, and cauda epididymis and identified a total of 725 genes. Overall, most immune-related genes are expressed in the caput epididymis, followed by the cauda and corpus, with most exhibiting significant downregulation in the post-pubertal caput, corpus, or cauda epididymis. Functional analysis suggested that significant enrichment of these genes was in pathways directly related to the make-up and function of the BEB [37,39], such as the tight junction, gap junction, adherens junction, ECM–receptor interaction, and regulation of actin cytoskeleton, as well as being associated with systemic immunosuppression [34], such as cytokine–cytokine receptor interaction and B/T cell receptor and Toll-like receptor signaling pathways. It is well recognized that the caput epididymis provides a tolerogenic environment, while the cauda epididymis favors pro-inflammatory conditions. Protecting spermatozoa from autoimmunity and defending them from pathogenic harm both rely on a well-controlled immune environment [40]. Thus, the reason for the higher number of immune-related genes in the caput and cauda epididymis of Tibetan sheep might be to protect spermatozoa from autoimmunity and defend them against pathogenic damage.

Additionally, we analyzed the expression patterns and functional characterization of eight known gene families (GPXs, DEFBs, LCNs, ILs, TLRs, TGFs, PRDXs, and AQPs) implicated in sperm maturation to identify potential candidates and their biological roles during maturation of spermatids of Tibetan sheep. Antioxidant activities can be found in genes belonging to the GPX family, and individual GPXs have specialized biological roles. To date, eight genetic isoforms of GPXs (GPX1-8) have been reported in mammals, and these genes are widely distributed in different organs of mammals [41,42]. Among the family of GPXs, the activity of GPX1-4 is dependent on selenium, of which GPX4 is an important structural protein that is very abundant in sperm mitochondria (about 50% of sperm mid-segment proteins), while when it is absent it leads to male infertility [43]. Another member of the GPX family, GPX5, which can protect sperm DNA from oxidative damage, is secreted by the principal cells within the epididymal epithelium and is highly expressed in the caput epididymis [42]. Similarly, we found that GPX5 exhibits high expression levels only in the caput epididymis, which was consistent with previous research in Small-Tail Han sheep that found a decreasing trend of GPX5 expression from the caput to cauda epididymis [3], suggesting that GPX5 is involved in the early stages of sperm maturation.

The epididymal microenvironment, including the BEB function, is maintained by the secretion of cytokines [1]. We evaluated the expression profiles of interleukins, Toll-like receptors, and transforming growth factors and found that the majority of all genes are downregulated in the post-pubertal epididymis, suggesting that most genes have an immunoprotective effect during the progressive maturation and motility of spermatozoa. DEFBs are a class of small cysteine-rich cationic peptides that serve antibacterial and anti-inflammatory activities and combine with sperm to control sperm maturation in the epididymis to ensure environmental stability [44]. Many DEFBs have been found in mammals where they form 4–5 syntenic gene clusters [45]. Almost all of the DEFBs are highly abundant in the epithelial cells in the male reproductive tracts, especially the epididymis [46]. Zhang et al. [47] showed that DEFB42, DEFB23, and DEFB26 are significantly more abundant in the caput and corpus epididymis, and both regions are known to be major contributors to sperm maturation. We found their homologs, DEFB33/36/107A/109/110/112/113/115/119/125/130/134, in the caput and corpus epididymis, and all of them showed highly upregulated expression levels at post-puberty, suggestive of roles in immunoprotection and maturation of spermatozoa. The lipocalin family participates in lipid transport and other biological functions, such as sperm maturation and storage and immune response [29]. Although Thimon et al. [11] found evidence of the existence of eight lipocalins in human epididymis, only LCN2 was abundant in the caput, whereas LCN6/8/10 were abundant in the corpus. This is not quite consistent with our study, where LCN6/8/10 were found to be highly present in the caput epididymis of Tibetan sheep, but is consistent with a study on pigs [48]. Notably, an interaction network among genes revealed that two TGFb isoforms (TGFB1 and TGFB2, well-known immune-homeostasis-related genes) and the receptor TGFBR2 were in the more central position of the network, participating in various cellular biological processes (such as cellular growth, proliferation, division, migration, morphogenesis, and junction) based on functional annotation. These results strongly suggested that these genes have multiple roles during sperm maturation and microenvironmental homeostasis in the epididymis of Tibetan sheep, but the mechanism by which they function in sperm maturation needs to be explored thoroughly.

## 5. Conclusions

In summary, we determined that the three conventional areas (caput, corpus, and cauda) of the epididymis of Tibetan sheep demonstrate significant variations in the expression of genes linked to their functions. Most genes exhibited stage–region-dependent expression changes and very active gene expression in the caput epididymis. Differential genes in each epididymal region of development participated in a wide variety of biological activities as well as related pathways, including growth and development, cell events, reproduction, metabolism, and immune function. The pre-pubertal epididymal regions showed very few and variable changes in gene expression, whereas changes in gene expression for the post-pubertal epididymal regions were dramatic, and specific genes were characterized by differential functions executed in the caput, corpus, and cauda epididymis for creating a microenvironment suitable for sperm development and storage as well as facilitating the maturation of spermatozoa. These results shed light on the spectrum of transcriptome changes that occur during epididymis development in Tibetan sheep, providing a foundation for further studies of the mechanism underlying sperm maturation in sheep.

## Data Availability

The raw RNA-seq data reported in this paper have been deposited in the Genome Sequence Archive in the National Genomics Data Center, China National Center for Bioinformation/Beijing Institute of Genomics, Chinese Academy of Sciences (GSA: CRA009752) that are publicly accessible at https://ngdc.cncb.ac.cn/gsa (accessed on 14 February 2023).

## Supplementary Materials

The following supporting information can be downloaded at: https://www.mdpi.com/article/10.3390/ani13091553/s1, Table S1: Primer information of mRNAs and miRNAs used for qPCR; Table S2: Summary of read numbers aligned onto the sheep reference genome; Table S3: List of differentially expressed genes associated with immunity.
