# Supplementary material for: Identification and Functional Assignment of Genes Implicated in Sperm Maturation of Tibetan Sheep
Source: Animals (Basel). 2023 May 6;13(9):1553. doi: 10.3390/ani13091553 (PMC10177108; doi:10.3390/ani13091553)
Supplement: Supplementary file 1 [file animals-13-01553-s001.zip › Table S1.pdf]

Table S1. Primer information of mRNAs and miRNAs used for qPCR.

Gene name	Primer sequence-Forward (5′-3′)	Primer sequence-Reverse (5′-3′)	Accession no.	Amplicon (bp)
NPC2	GAATGTGAGCCCATGTCCCA	CCATGTACCACAGCCTTGCT	XM_012182049.3	117
PAX2	GTTGTGACCGGTCGTGACAT	TGCTGAATCTCCAAGCCTCG	NM_001178052.1	184
PRDX6	TCGGGACTTTACCCCAGTGT	CTGTGGGCTCTTCACCATTG	NM_001280704.1	167
ADAM7	AGCAAGTTCAAAGCCCACCT	TGGTTCTTCGCTGCTGATGT	XM_042243125.1	129
AQP9	GTAGCAGCCACCATGTTCCT	ATTCCCAGAGAGGAGGCGAT	XM_004010567.5	131
GJA1	TGCCAATGTGGACATGCACT	AAGAAGGCCACCTCGAAGAC	XM_004011159.5	150
ELSPBP1	GCAGCAGTGGAAATACTGCG	CCATGTTCTCCGTGGTTGGA	XM_027978548.2	152
CLDN7	CCGTTGGTCCCCATGAATGT	CAGCTTTGCTCTCACTCCCA	NM_001185018.1	130
HSPA2	TCGGAAGATGAGGCCAATCG	CGCTAATCTTGCCCCTCAGT	XM_004010719.5	112
ODF1	TGACGTACTCCTACGGGCTC	AACCGGCTCCCACAAGGATA	XM_004011803.5	199
GLB1	CTGAACGCCATCCAGACGTA	CTCCAACAGCCAAGCAGGTA	XM_015102219.3	195
GPX5	AATGTGGCCACCTATTGTGGT	TGGTTACACGGAAATCCCAACA	NM_001267883.1	107
β-actin	ATATTGCTGCGCTCGTGGTT	GTTGGTGACAATGCCGTGCT	NM_001009784.3	224
