# Supplementary material for: Identification and Functional Assignment of Genes Implicated in Sperm Maturation of Tibetan Sheep
Source: Animals (Basel). 2023 May 6;13(9):1553. doi: 10.3390/ani13091553 (PMC10177108; doi:10.3390/ani13091553)
Supplement: Supplementary file 1 [file animals-13-01553-s001.zip › Table S2.pdf]

Table S2. Summary of read numbers aligned onto the sheep reference genome.

Sample	Total	Unmapped (%)	Unique_Mapped (%)	Multiple_Mapped (%)	Total_Mapped (%)
Cp-1Y-1	62514808	7274698 (11.64%)	52360674 (83.76%)	2879436 (4.61%)	55240110 (88.36%)
Cp-1Y-2	51423816	5928112 (11.53%)	43054539 (83.72%)	2441165 (4.75%)	45495704 (88.47%)
Cp-1Y-3	42833866	5277105 (12.32%)	35661218 (83.25%)	1895543 (4.43%)	37556761 (87.68%)
Cp-1Y-4	44006918	5066313 (11.51%)	37026148 (84.14%)	1914457 (4.35%)	38940605 (88.49%)
Cp-3M-1	64756588	7452496 (11.51%)	53193684 (82.14%)	4110408 (6.35%)	57304092 (88.49%)
Cp-3M-2	52551240	6084114 (11.58%)	43384027 (82.56%)	3083099 (5.87%)	46467126 (88.42%)
Cp-3M-3	56791208	6206144 (10.93%)	47084879 (82.91%)	3500185 (6.16%)	50585064 (89.07%)
Cp-3M-4	43496810	4710161 (10.83%)	36338012 (83.54%)	2448637 (5.63%)	38786649 (89.17%)
Cr-1Y-1	58521938	6645135 (11.35%)	48925020 (83.60%)	2951783 (5.04%)	51876803 (88.65%)
Cr-1Y-2	49913278	5688678 (11.40%)	41375669 (82.90%)	2848931 (5.71%)	44224600 (88.60%)
Cr-1Y-3	54881564	6316124 (11.51%)	45671874 (83.22%)	2893566 (5.27%)	48565440 (88.49%)
Cr-1Y-4	59426474	6548401 (11.02%)	49731776 (83.69%)	3146297 (5.29%)	52878073 (88.98%)
Cr-3M-1	53288852	6539519 (12.27%)	43472815 (81.58%)	3276518 (6.15%)	46749333 (87.73%)
Cr-3M-2	59370152	7629424 (12.85%)	48340956 (81.42%)	3399772 (5.73%)	51740728 (87.15%)
Cr-3M-3	40833528	5263799 (12.89%)	33403273 (81.80%)	2166456 (5.31%)	35569729 (87.11%)
Cr-3M-4	53387928	6404579 (12.00%)	44167474 (82.73%)	2815875 (5.27%)	46983349 (88.00%)
Cu-1Y-1	47851492	6133777 (12.82%)	39532932 (82.62%)	2184783 (4.57%)	41717715 (87.18%)
Cu-1Y-2	47950258	5551004 (11.58%)	40104196 (83.64%)	2295058 (4.79%)	42399254 (88.42%)
Cu-1Y-3	56745986	6527005 (11.50%)	47448971 (83.62%)	2770010 (4.88%)	50218981 (88.50%)
Cu-1Y-4	55732240	6153554 (11.04%)	47050498 (84.42%)	2528188 (4.54%)	49578686 (88.96%)
Cu-3M-1	46813082	5589854 (11.94%)	38620217 (82.50%)	2603011 (5.56%)	41223228 (88.06%)
Cu-3M-2	42502660	4774938 (11.23%)	35173597 (82.76%)	2554125 (6.01%)	37727722 (88.77%)
Cu-3M-3	45209036	4860421 (10.75%)	37820107 (83.66%)	2528508 (5.59%)	40348615 (89.25%)
Cu-3M-4	56578100	6364218 (11.25%)	47040229 (83.14%)	3173653 (5.61%)	50213882 (88.75%)

Note: Cp, Cr, and Cu denote the caput, corpus and cauda epididymis, respectively; 3M and 1Y indicate three-month-old and one-year-old, respectively.
